# 

**Published:** 1897-04-03

**Authors:** 


### The Hospital

A JOURNAL OF

THE MEDICAL SCIENCES AND HOSPITAL ADMINISTRATION,

INCLUDING

A SPECIAL SECTION FOR NURSES.

EDITED BY

SIR HENRY BURDETT, K.C.B.,

AND

SOLOMON C. SMITH, M.D.

IN TWO VOLUMES ANNUALLY.

VOLUME XXII.

APRIL TO SEPTEMBER, 1897.

SURGEON GEN'L'S OFFICE,
158765
LIBRARY.

London:

PUBLISHED BY THE SCIENTIFIC PRESS (LIMITED), AT THE HOSPITAL BUILDING,
28 & 29, SOUTHAMPTON STREET, STRAND, W.C.

MDCCCXCVII.

W1 HO 69X

### INDEX TO VOL. XXII., 1897.

*** Articles under the general heading *Medical Progress and Hospital Clinics* are distinguished by the initial (C.) for *Hospital Clinics*, and by (P.) for the rest of the series. (W.) distinguishes the *Institutional Workshop* articles, and (A.H.) the notes under the title *Around the Hospitals*.

Abdomen, Gunshot Wounds of (P.), 200
Abdominal Hydatids, Intra-peritoneal Treatment of (P.), 200
Abdominal Surgery—A Manœuvre, 179
Abscess, Cerebral, Diagnosis of (P.), 129
Abscess, Traumatic Cerebral (P.), 98
Achillodynia (P.), 373
Acne. *See p.* 75; (P.), 132
Aconite Treatment. *See p.* 33
Adaptation in Pathology (C.), 300
Adelaide Hospital, South Australia, The, 89
Adelaide Hospital and the B.M.A., The, 242
Adherent Placenta (P.), 78
Adulteration of Food, The, 279
Aerial Therapy (P.), 182
Alloa, New Accident Hospital at (W.), 206
American Health Resorts, 261
Anæmia, Pernicious (P.), 442
Anæsthesia in Labour (P.), 78
Anæsthetics, Deaths Under (C.), 334
Anæsthetics, German Statistics Regarding, 98
Anæsthetics, More Deaths from, 55
Anastomosis, Intestinal (P.), 410
Aneurism and Atheroma (P.), 355
Angina Epiglottidea Anterior (P.), 375
Anterior Tibial Curve (C.), 214
Antiphthisin (P.), 215
Antiseptics (P.), 13, 393
Antiseptics in Medical Cases (W.), 204
Anti-toxic Serum, Asylums Board Report on (C.), 142
Anti-toxic Serum, The Plague and (C), 424
Anti-toxin, Nephritis and (P.), 323
Anti-tuberculous Serum (P.), 196
"Anti-vivisection" Hospital, An, 173
Aortic Stenosis (C.), 266
Aphasia (P.), 76
Apoplexy (P.), 78
Apothecaries' Hall, An Opportunity for, 139
Appendicitis (P.), 250, 443
Appendicitis and Peritonitis (C.), 407
Army Medical Service, The, 21
Around the Hospitals (A.H.), 256, 362, 381, 451
Arterial Hypermyopathy, Idiopathic (P.), 354
Ascites (C.) *See p.* 301
Asthenopia and Spectacles (C.), 247
Asylum Construction (W.), 186
Asylum Officials and Their Pensions, 366
Asylums Board, The Enlarged Functions of the, 23
Atheroma and Aneurism (P.), 355
Athletes—Are Athletes Healthy? 421
Bacteriological Conditions present in Ulcers Treated by Oxygen (C.), 93
Bacteriological Diagnosis, Pitfalls in (C.), 59
Bacteriological Problems (C.), 407
Baldness and Dandruff (C.), 301
Baldness, The Cause of, 3
Bare Feet, 385
Barrack Schools, Mr. Ernest Hart and the, 71
Bearsden Convalescent Home (W.), 202
Bedminster Branch of the Bristol Dispensary (W.), 188
Birmingham General Hospital (A.H.), 256
Birmingham General Hospital, The New (W.), 272
Blackpool, The Victoria Hospital (W.), 399
Blackwall Tunnel from a Medical Point of View, The (C.), 126
Bladder, A Case of Rupture of the (C.), 388
Blount, Commander (Obituary), 171
Board Schools and Fevers, 436
Bolton Infirmary (A.H.), 381
Bones and Joints, Diseases of (P.), 31
Book World of Medicine, The (*Books, Magazines, and Pamphlets reviewed, or notices and articles relating to* :—
Allbutt. A System of Medicine, 447
Baily. Oban, 377
Beale. *See Jaruntowsky*
Benham. *See Brouardel*
Bishop. Diseases of the Ear, Nose, and Throat, 219
Bradshaw. Dictionary of Mineral Waters, &c., 288
Brouardel. Death and Sudden Death, 19
Burdett. Hospitals and Charities, 219
Clark. Clinical Manual of Mental Disease, 271
Davidson. Sight Testing for the G.P., 50.
Donald. Municipal Year Book for 1897, 201
Donald. The London Manual, 1897-8, 359
Franzenbad—An Austrian Health Resort, 377
Hart. Sanitation and Health, 165
Hawthorne. Forensic Medicine and Toxicology, 33
Hawtrey. The Co-Education of the Sexes, 149
Hendley. *See Hart*
Holthouse. Convergent Strabismus and its Treatment, 413
Jackson. Left-hand Writing, 377
James. Royal Infirmary Cliniques, 133
Book World of Medicine (*continued*).
Jaruntowsky (trans. by Beale). Private Sanatoria for Consumptives, 133
Johnson. Swedish System of Physical Education, 359
Kenny. Diet Chart and Alimentary Index, 377
Kirstein (trans. by Thorner). Autoscopy of the Larynx and Trachea, 271
London Health Laws, 201
McGillicuddy. Functional Nervous Disorders in Women, 19
Marcet. A Contribution to the History of Respiration in Man, 359
Martin and White. Genito-Urinary Surgery and Venereal Diseases, 201
Maxwell. Notes on Practical Sanitary Science, 377
Medical Annual, 1897, 19
Moncrieff. Black's Guide to Bournemouth and the New Forest, 133
Moor. *See Pearmain*
Morgan. The Climate of Bournemouth in Relation to Heart Disease, 165
Napier. The Menopause and its Disorders, 81
Observer, An. Sanitary and Social Questions of the Day, 359
Owen. Surgical Diseases of Children, 307
Paget. Wasted Records of Disease, 50
Pearmain and Moor. Aids to the Study of Bacteriology, 81
Ranney. Eye-Strain in Health and Disease, 433
Reynolds-Ball. Mediterranean Winter Resorts, 133
Roberts. Digestion and Diet, 413
Sherard. The White Slaves of England, 235
Sibbald. On the Plans of Modern Asylums for the Insane Poor, 133
Sladen. Who's Who, 1897, 185
Smith, Henry. A Plea for the Unborn, 149
Smith, J. B. Quantitative Estimation of Urine, 99
Snell. Compressed Air Illness, or So-Called Caisson Disease, 185
Squire. The Treatment of Lupus, 185
Stedman. Twentieth Century Practice, 185
Thorington. Retinoscopy, 307
Thorner. *See Kirstein*
Tirard. Diphtheria and Anti-toxin, 19, 413
Toxicology, 149
Walsh. Excretory Irritation, 271
White. *See Martin*
Yorke-Davies. Homburg and Its Waters, 377
Brain, Foreign Body in the (P.), 98
Brain, Gummata of (P.), 131
Brain, Tuberculoma of (P.), 131
Brain, Tumours of the (P.), 62, 130
Brain Surgery (P.), 129, 146
Brain Surgery, Hæmorrhage in (P.), 147
Bravery and its Rewards, 315
British Medical Association and its Helpless Members, 279
Bromides. What Happens to All the Bromides? (C.), 265
Bruise, The History of a, 123
Burial Insurance, The Problems of, 317
Caird Hospital for Women, Dundee (W.), 85
Calculus, Renal (C.), 10
Calf Lymph, Glycerinated (C.), 178
Calomel (P.), 354
Cancer. "Remedies for Cancer" (C.), 43
Cape Town Institutions, Some (W.), 254
Carcinoma, Operations for (P.), 232
Cardiac Disturbance in Relation to Dyspepsia (C.), 407
Cardiac Therapeutics (P.), 339
Central Hospital Board for London, A, 71
Cerebellum, Glio Sarcoma of the (P.), 131
Cerebral Abscess, Traumatic (P.), 98
Cerebral Lesions and Intestinal Disorders (P.), 305
Cerebral Localisation (P.), 147
Charity Organisation Society and the Hospitals (W.), 134, and *See p.* 165
Chemistry and Pathology (C.), 424
Chemists' Exhibition, The, 358
Chicken Pox (P.), 324
Child. That Mystery the Child, 263
Child Life Under Queen Victoria, 5, 25, 41, 57, 73
Children, Diseases of (P.), 285, 304, 324
Children, The Morals of, 361
Cholera Nostras Asiatica (C.), 282
Chloroform, Deaths from, 175
Chloroform, The Administration of, by Regulating Inhaler (C.), 371
Chloroform Syncope, The Cause and Treatment of (C.), 75
Church and Sanitation, 436
Clinical Research Association, 25
Clinical Society (C.), 7, 42
Club Foot, Paralytic, Treatment of (P.), 357
Congresses, Notes from the (C.), 406
Congresses, The, 402
Construction Notes (W.), 18, 85, 188, 205
Convalescent Home, Grange-over-Sands, Lancashire (W.), 34
Co-operation and Charity, 91
Co-operative Celibacy, 437
Cord, Wounds of the (P.), 63
Correspondence. *See Editor's Letter Box*
Cosy Houses, 403
Cottage Hospital Administration (W.), 399
Cottage Hospital, The Furnishing and Appliances of a (W.), 65
Cottage Hospital Trustees. *See p.* 171
Court Appointment, A, 314
Criminals, Young, at Home and Abroad, 367
Cry, A Good, 315
Cystic Tumours of Ovary and Broad Ligament, Rate of Growth of (P.), 79
Dairymaid, The Education of the, 299
Dandruff, Baldness and (C.), 301
Death by Fire, 106
Death in the Bath, 89
Death Rates and Health, 297
Dentist, The Qualified, 397
Dermatitis, X-Ray (P.), 148
Dermatology (P.), 15
Dermatology, Periscope of (P.), 115, 132, 148, 163
Despot, The Coming, 55
Diabetes (P.), 426
Diabetes, The Dietetic Treatment (C.), 440
Diagnosis, Pitfalls in Bacteriological (C.), 59
Diagnosis, Treatment before (C.), 58
Digestive Organs, Diseases of the (P.), 94, 112, 230, 266
Digitalis and Blood Pressure, (P.), 340
Diphtheria (P.), 302, 322
Diphtheria and Anti-toxin. *See p.* 200
Diphtheria and Sanitation (C.), 440
Dispensary Abuse, American Action in Regard to, 123
Disinfecting Apparatus (W.), 238
Disinfection (C.), 111
Disinfection of Infected Rooms, 191
Divining Rods Again! 155
Doctor or Not Doctor, 347
Doctors in Camerâ, 296
Dundee, Proposed House of Mercy (W.), 188
Dundee Royal Infirmary (W.), 85
Ear Disease, The Question of Operation for Non-Suppurative (C.), 110
East Riding Lunatic Asylum (W.), 186
Eclampsia, Puerperal (P.), 63
Editor's Letter-Box, 33, 49, 99, 135, 151, 165, 200, 222, 257, 289, 327, 399, 450.
Education of the Judgment, 122
Electricity and Dust (C.), 247
Empyema, Clinical Lecture on the Treatment of (C.), 389
Endocarditis (C.), 195
Endocarditis, Malignant (P.), 320
English Doctors Abroad, 223
Epidemic Hospital at Inverurie (W.), 18
Epidemic Infantile Paralysis (C.), 264
Epidemiological Society (C.), 6, 93, 158
Epilepsy. *See p.* 46; (P.) 77
Epileptics, The Colony for, 105
Ethmoidal Sinus (P.), 287
Execution by Electricity, 348
Fæcal Fistula (P.), 411
Femoral Hernia, The Radical Cure of (C.), 441
Fever Hospitals and the Profession, 313
Fevers (P.), 145, 180, 336.
Fevers, The Specific, and their Complications (P.), 304
Fibro-Myomata of the Uterus (C.), 248
Finger as a Tourniquet, The Aseptic (C.), 158
Fistula, Fæcal (P.), 411
Forceps, The Abuse of the, 419
Fracture, The Treatment of, 260
Fractures and Dislocations (P.), 14, 411
Fractures, Laminectomy for Simple (P.), 63
Fractures, Some Points in the Treatment of (C.), 335
Fragilitas Ossium (P.), 324
Fringe of the Profession, The, 39
Frontal Sinus (Rhinology P.), 270
Fumigation of an Ambassador, The, 87
Gas, The Enrichment of, 297
Gastric Spasm, Congenital (P.), 324
Gastric Ulcer, Perforation, Hæmorrhage (P.), 428
Gastro-enterostomy (P.), 429
Gastrostomy (P.), 428
General Medical Council, The Session of the (C.), 159
General Medical Council, The Vacancy in the, 436
Glasgow, New Pathological Institute at (W.), 205
Glio-sarcoma of the Cerebellum (P.), 131

Gloucester General Infirmary (A. H.), 451
Goitre, Exophthalmic. *See p.* 46
Gonorrhœa in Women (P.), 47
Gout and Rheumatism (P.), 12, 29
Gout, The Treatment of, by Hot Dry Air (C.), 264
Grange-over-Sands Convalescent Home (W.), 34
Gratitude to the Hospitals, 235
Grocers and Adulteration, 331
Growth and Scope of Voluntary Hospitals in London (W.), 290, 308, 325, 341, 361, 378, 398, 414, 431, 448
Guiacolate of Piperidine (P.), 183
Guy's Hospital New Medical School (W.), 253
Gynæcology, Progress in (P.), 47, 63
Hæmatocele, Pelvic (C.), 213
Hæmorrhage, Ante Partum (P.), 79
Hair Dyes. By Whom are Hair Dyes Used? 225
Hair Dressing, The Dangers of, 295
Harrogate Royal Baths, The, 315
Harveian Society, The (C.), 26, 92
Head Injuries (P.), 97
Health of the Army in India, The (C.), 318
Healthy Little Town, A (C.), 74
Heart and Circulation, Diseases of the (P.), 320, 338, 354
Heart Wall, Syphilitic Disease of the (P.), 321
Hereford General Infirmary (A. H.), 256
Hernia, Femoral. The Radical Cure of (C.), 441
Herpes Zoster (P.), 148
Hodgkins's Disease (P.), 443
Holidays, 402
HOSPITAL, THE, to its Readers, 2
Hospital Construction (W.), 34, 82, 100, 202, 236, 253
Hospital Festivals, Meetings, &c. (W.), 17, 35, 51, 103, 119, 134, 150, 168, 203, 292, 310, 327, 345
Hospital, Impressions of a Russian, 449
Hospital Reports (W.), 221
Hospital Saturday Fund's System of Distribution (W.), 16, 49
Hospital Sunday and the Queen's Commemoration (W.), 220
Hospital Sunday Fund, Metropolitan (W.), 342
Hospital Sunday Fund, The Distribution of the, 329
Hospital. *See also Cottage Hospital*
Hospitals and their Supporters in South London (W.), 16
Hospitals and Vivisection, The (W.), 117
Hospitals, Charity, and Organisations, 435
Hospitals, In the, Sixty Years Ago, 157
Hospitals, Isolation (P.), 305
Hospitals. *See Growth and Scope of Voluntary, and Voluntary Hospitals*
Hotel, Death in an, 155
Hotel, The Poor Man's, 155
Housing of the Working Classes Act, The, 121
Hygiene, Industrial (P.), 162
Hygiene, School (P.), 162
Hysterical Mutism (P.), 77
Icthyol (P.), 15
Infancy, A Rare Fatal Disease of (P.), 286
Infantile Paralysis, Epidemic (C.), 264
Infantile Paralysis (P.), *See p.* 357
Infants, The Feeding of (P.), 285
Infection, The Conveyance of, 1
Inquests, Improved Accommodation for, 301
Insane. The Story of the Insane from Year to Year (W.), 18, 67, 84, 102, 187, 236, 255, 275, 291, 309, 345, 362, 379, 415, 432, 451
Insanity (P.), 78
Insanity, Delusional (C.), 9
Insanity in Prisons, 421
Intestinal Anastomosis (P.), 410
Intestinal Disorders and Cerebral Lesions (P.), 305
Intestinal Obstruction (P.), Acute, Chronic, 392
Intestinal Obstruction, The Treatment of (C.), 230
Intestines (Diseases of the Digestive Organs) (P.), 113, 232, 249
Intubation (P.), 323
Intussusception (P.), Acute, Chronic, 392
Inverurie, Epidemic Hospital at (W.), 18
Isolation Hospitals (P.), 305
Jubilee Eviction, A, 175, 289
Jubilee Stamps, What to do with the (W.), 203
Kneippism, 437
Knock-Knee (C.), 213
Kocher's Method (P.), 46
Laminectomy for Simple Fractures (P.), 63
Laryngeal Paralysis (P.), 356
Laryngitis, Scarlatinal (P.), 375
Laryngitis, Varicellous, 375
Larynx, Toxic Paralysis of the (P.), 375
Lateral Ventricle, Puncture of the (P.), 147
Laundry Construction (W.), 238
Lead Poisoning (P.), 62
Leeds General Infirmary (W.), 236, 239
Leprosy (P.), 15, 115
Liberty and Sanitation, 421
Liver, Diseases of the (P.), 95, 267
Lockjaw, Spasmodic (P.), 47
Locomotor Ataxy (P.), 61
Long Bones, Congenital Defects of the, 356
Longevity, The Art of, 365
London General Hospitals, and the Central Hospital Board, The (W.), 187
London, The Port of, 55
Lower Extremity, Surgery of the (P.), 429
Lumbago (C.), 178
Lumbar Puncture (P.), 114, 324
Lunacy, The Increase of, 281
Lunatics in Unlicensed Houses, 99
Lungs, Diseases of the (P.), 182, 196, 215, 268
Luxor Hospital for Natives (W.), 82
Madmen, The Making of, 23, *and see p.* 99
Malaria (P.), 337
Malaria and its Mode of Access (C.), 58
Manual Training in Surgery, 70
Mastoid, Operations on the (P.), 130
Maxillary Sinus (P.), 252
Measles, The Notification of, 38
Meckel's Diverticulum (P.), 411
Medical Acts, The Defects of the, 403
Medical Certificates and Workmen's Compensation, 261
Medical Council and the Apothecaries' Hall of Ireland, The, 37
Medical Dogmatism, 349
Medical Education. *See p.* 151
Medical Education in America, 165
Medical Education in Scotland, 397
Medical Officers and their Reports, 297
Medical Practice, The Perils of, 277
Medical Practice. The State Control of (C.), 26
"Medical Press, The," and The Hospital Sunday Fund, 417
Medical Schools of the Metropolis, The, 395
Medical Science and Anti-Vivisectionists. *See p.* 222
Medical Science in the Queen's Reign, 189
Medical Society, The Oration of the (C.), 143
Medical, Surgical, and Hygienic Exhibition, The, 311
Medical Teaching in Germany, 107
Medicine as a Profession, 383
Medicine as a Study, 384
Medicine, The Study of, in London, 395
Medico-Legal Intelligence, 221
Medico-Psychological Society and Men Nurses (W.), 51
Menopause, Neuroses of the, 111
Mental Nurses and the Medico-Psychological Society (W.), 51
Mental Nurses and the R.B.N.A., 49
Metatarsalgia, or Morton's Disease (P.), 374
Metropolitan Asylums Board, 224
Middlesex Hospital Convalescent Home, Clacton-on-Sea (W.), 100
Midwifery Nurses, 261
Milk (P.), 305, 445
Mitral Disease (P.), 338
Modern Sociology, 91, 109, 125, 141, 157, 177, 193, 211, 227, 245, 263, 281, 299, 317, 333, 351, 369, 387, 405, 423, 439
Morton's Disease, or Metatarsalgia (P.), 374
Motor Innervation (P.), 355
Muscle-Transplantation (P.), 357
Muscular Atrophy (C.), 265
Mutism, Hysterical (P.), 77
Mutual Admiration Societies (C.), 424
Muzzling Order and its Effects, 53
Nasal Obstruction (P.), 287
Negro, The Decadence of the 54
Nephritis and Anti-Toxin (P.), 323
Nerve, Cervical Sympathetic, Resection of (P.), 46
Nerve Grafting (P.), 46
Nerves, Spinal, Intradural Section of, for Neuralgia (P.), 46
Nervous Diseases, Some Resources in the Diagnosis of (C.), 128
Nervous System, A New Light on the (C.), 179
Neuritis, Compression in, Traumatic (P.), 46
Neurology (P.), 61, 76
Neuroses, Hereditary, in Children, 109, 125, 141, 177, 193, 211, 227
Neuroses of the Menopause (C.), 111
Neurotic Symptoms of Uricacidæmia (P.), 324
New Appliances and Things Medical, 48, 64, 80, 164, 184, 218, 252, 324, 340, 376, 394, 430, 446
New Growths (P.), 355, 390, 391
Nitro-Glycerine (P.), 340
No Creed for the Criminal, 349
Nomenclature of Diseases, The 23
North-Eastern Hospital for Children (A. H.), 362
Notes and News, 4, 24, 40, 56, 72, 90, 108, 124, 140, 156, 176, 192, 210, 226, 244, 262, 280, 298, 316, 332, 350, 368, 386, 404, 422, 438
Notes of Hospital Clinics (C.), 265, 335, 352, 370, 388
Nursing in Workhouses (W.), 102, *and see pp.* 135, 151
Œsophagus and Stomach, Diseases of the (P.), 428
Œsophagus, Cancer of the (P.), 428
Œsophagus, Cicatricial Stricture of the (P.), 428
Œsophagus, Foreign Bodies in the (P.), 428
"Off his Game," 208
Oils and Explosions, 335
Optic Neuritis in Typhoid Fever (C.), 195
Orthopædic Progress (P.), 356, 373
Osteomyelitis, Vertebral (P.), 115
Osteo-plastic Resection of the Tarsus (P.), 357
Osteopsathyrosis, Idiopathic (P.), 324
Oxygen Home, The (W.), 221, 257
Pamphigus (P.), 132
Pancreas, Diseases of the Digestive Organs (P.), 112
Paquin's Serum (P.), 215, 250
Paralysis, Epidemic Infantile (C.), 264
Paralytic Club-Foot, Treatment of (P.), 357
Park Hospital, Hither Green, The (W.), 205
Parks and Poor Rates, 279
Patellæ, Congenital Absence of (P.), 357
Pathology, Adaptation in (C.), 300
Pay System in Hospitals, The (W.), 83
Pelvic Hæmatocele (C.), 213
Peritoneal Infection (P.), 199
Peritoneum, Diseases of the Digestive Organs (P.), 113, 266
Peritoneum, Absorption of Fluids by the (P.), 199
Peritoneum, Drainage of the (C.), 407
Peritonitis and Appendicitis (C.), 407
Peritonitis, Tuberculous (P.), 199
Perth Royal Infirmary (A.H.), 363
Pharyngitis and Tonsillitis (P.), 355
Pharynx (P.), 355
Pharynx and Larynx, Diseases of the (P.), 355, 375, 390
Phimosis (P.), 59
Picric Acid (in Dermatology, P.), 163
Pistol-shot Injuries (P.), 97
Placenta, Adherent (P.), 79
Plague, 71, (P.) 181
Plague and Anti-toxic Serum, The (C.), 424
Plague. Is the Plague Contagious? 327
Plague, The, on the Red Sea, 225
Pneumectomy (P.), 268
Police Cases at Hospitals, 243
Pollution of Rivers, The, 89
Poor Law Guardians, The Responsibilities of, 209
Poor Man's Home, The, 351, 369, 387, 405, 423
Poor Man's Lawyer, The, 91
Population Statistics for 1895, 69
Potts' Disease, The Surgical Treatment of (P.), 160
Powder and Paint, 139
Practical Departments (W.), 18, 171, 238, 417
Practical Points (W.), 221, 310
Prince of Wales's Hospital Fund for London, The (W.), 166, 275
Prince of Wales's Hospital Fund Stamps, The, 108, 134, 166
Professional Badges, 139
Progress in State Medicine (P.), 32
Pruritus (P.), 15
Public Baths and Personal Cleanliness, 403
Public Gardens, 209
Puerperal Eclampsia (P.), 63
Pyæmia, Otogenous (P.), 130
Pylorus, Strictures of, Non-Malignant (P.), 429
Quackery, The Suppression of, 153
Queen, The Health of the, 191
Queen's Hospital (A.H.), 381
R.B.N.A. and Mental Nurses, The, 49
Radiography and Surgery (P.), 30
Rare Fatal Disease of Infancy, A (P.), 286
Rather Premature, 39
Refuse Disposal (P.), 163
Renal Calculus (C.), 10
Renal Medicine (C.) 44, (P.) 372, 409
Reproduction in Plants, Modes of, 55
Resection of Lung (P.), 268
Resection, Temporary, of the Skull (P.), 147
Rheumatic and Atheromatous Valve Lesions (P.), 338
Rheumatism and Gout (P.), 12, 29
Rhinitis, Atrophic (P.), 233
Rhinitis, Hypertrophic (P.), 252
Rhinology (P.), 233, 252, 269, 287
Rickets, Late (P.), 324
Rickets, Treatment of Deformities Caused by (C.), 195, 213
Robertson, C. Lockhart (Obituary), 150
Roentgen Rays (and Heart Disease, P.), 320
Roentgengraphy (P.), 391
Röntgen Society, The, 211
Rosebery House, 225
Royal College of Surgeons, The Council of the (W.), 203
Royal Hants County Asylum (A. H.), 263
Royal Medical and Chirurgical Society (C.), 7, 42, 74, 110, 142, 178
Sacro-iliac Disease (P.), 161
St. John Ambulance Association (P.), 326
Salisbury Infirmary (A. H.), 451
Sanitary Administration in America (C.), 424
Sanitary Pharisees, 39
Sanitation and Morality, 123
Sanitation and Superstition, 241
Sanitation for Scotland, 137
Scabbing, Treatment of Ulcers by (C.), 159
Scarlet Fever (P.), 336
Schaw Convalescent Home, Bearsden (W.), 202
School Children as Wage Earners, 349
School Inspection (P.), 32
Sclerosis, Disseminated (P.), 61
Sclerosis, Disseminated—*Sclérose en Plaques* (P.), 375
Scoliosis, The Value of an Adapted Bicycle in (P.), 356
Scraps and Gleanings, 20, 52, 64, 221, 239, 257, 274, 293, 345, 363, 381, 451
Scurvy, 107
Sea-sickness, 419
Seaside Dangers, 331
Seborrhœa and Acne, The Microbes of (C.), 75
Sensational Paragraphs, The Truth of, 351
Septum, Diseases of the (P.), 287
Serum, Anti-Streptococcic, The Surgical Uses of (C.), 27

Serum, Asylums Board Report on Anti-toxic (C.), 142
Serum, Paquin's (P.), 215
Serum Test for Typhoid Fever, The (C.), 8
Serum Therapy (Diphtheria, P.), 302, 322
Serum Treatment and its Limitations, 139
Sewage, Purification of (P.), 32, *and see pp.* 48 *and* 50
Shelters for the Homeless, 154
Ship-surgeon, The, 861
Singer's Nodules (P.), 391
Sinus, Ethnoidal (P.), 287
Slaughter Houses, The Question of the, 278
Small-Pox (P.), 337
Smith, James Greig (Obituary), 171
Societies and Transactions (C.), 300
Sociology. *See* Modern Sociology
Solitary Confinement and Insanity, 333
Specialist and the Practitioner, The, 22
Spectacles and Asthenopia (C.), 247
Spinal Caries: Forcible Reduction of the Curve (P.), 356
Spine and Cord, Injuries of the (P.), 62
Spine, Rickety Curves of the (C.), 214
Spleen (Diseases of the Digestive Organs, P.), 113, 267
Spleen, Enlarged, Lecture on (C.), 370
Sprained Ankle (C.), 353
Stenosis, Aortic (C.), 266
Stomach. *See also* Œsophagus
Stomach, Cancer of the, Resection (P.), 429
Stomach, Diseases of the (P.), 94
Stomach, Foreign Bodies in the (P.), 429
Stomach, Hour-glass Contraction of the (P.), 429
Stomach, Operations on the (P.), 428
Story of the Insane. *See* Insane
Street Collections, 257
Stricture of the Urethra, Treatment of (P.), 216
Student of Medicine, The, 20, 36, 68, 86, 104, 152, 172, 206, 239, 2[illegible]8, 276, 294, 312, 346, 364, 382, 400, 418, 434, 452
Sub-peritoneal Tissues (P.), 200
Sudden Death, The Causes of (C.), 229
Sugar Tests for Diabetes (P.), 426
Surgery, Abdominal, 179, 391, 410, 443
Surgery, Brain, 129, 146
Surgery, General, 15, 29, 393, 411, 429
Surgery, Genito-urinary, 198, 216
Surgery of the Nerves, 46
Surgery of the Peritoneum, 199
Surgery of the Rectum, 232
Surgery, Spine, 62, 114, 161
Surgery of the Ureter, 217
Surgery, Drainage in Abdominal (P.), 200
Surgical Aid Society (W.), 35
Surgical Treatment of Lung Disease (P.), 251
Synovitis of the Knee-joint, The Treatment of (C.), 143
Syphilis, Congenital (P.), 286
Syringomyelia (P.), 61
Tap or to Explore, To (C.), 301
Tarsus, Osteo-Plastic Resection of the (P.), 357
Technical Education, 207
Technical Education in London, 245
Temperature of the Body: The Means by which it is Maintained (C.), 194, 212, 228, 246
Temporal Region, Pulsating Tumour of the (P.), 131
Tendon-grafting, &c. (P.), 357
Testicle, Diseases of the (P.), 217
Tetanus (P.), 337, (C.), 370
Tetany (P.), 305
Theobromine (P.), 355
Tonsil, Lymphangioma of the (P.), 355
Tonsillitis and Pharyngitis (P.), 355
Trachea (Diseases of the Pharynx and Larynx, P.), 391
Trade-Like Movements following Head Injuries (P.), 97
Traumatic Cerebral Abscess (P.), 98
Traumatic Meningococle (P.), 98
Tricycles, A Good Word for, 331
Tuberculin Injections (P.), 183
Tuberculin, The New (P.), 197
Tuberculosis (P.), 182, 196, 250, 251 (C.), 407
Tuberculosis of the Skin (P.), 148
Tuberculous Peritonitis (P.), 199
Tumour, Fæcal (P.), 200
Tumours (Dermatology, P.), 163
Typhoid Fever. *See p.* 195
Typhoid Fever in Villages, 107
Typhoid Fever, Serum Test for (C.), 8
Typhoid Fever, Water Supply and, 174
Typhoid Fevers (P.), 145, 181
Typhoid Ulcers, Perforating (P.), 391
Typhus Fever (C.), 334
Ulcers of the Leg, Treatment of by Scabbing C.), 159
Ulcers, Perforating Typhoid (P.), 391
Ulcers Treated by Oxygen. *See p.* 93
Umbilical Sepsis (C.), 158
Upper Extremity, Fracture of the (P.), 411
Ureter, Surgery of the (P.), 216
Urethra, Stricture of the (P.), 216
Uricacidæmia, Neurotic Symptoms of (P.), 324
Urology (P.), 409
Uterus, Modern Views as to Fibro-myomata of the (C.), 248
Uterus, Vibratory Massage of the (C.), 59
Vaccination, An Encouragement to, 385.
Valve-Lesions, Rheumatic and Atheromatous (P.), 338
Vasectomy, Clinical Remarks on (C.), 8
Venice Convention, The, 3
Verminous Persons, 259
Vertebral Osteomyelitis (P.), 115
Vesical Calculus (P.), 198
Vestries, In Praise of, 367
Vital Statistics of Block Dwellings, The, 243
Vivisection and the Hospitals (W.), 117, and *see p.* 222
Voluntary Hospitals. *See* Growth and Scope, &c.
Voluntary Hospitals, The Rating of (W.), 416, 450
Vulvo-Vaginitis, Infectious (P.), 286
Wakefield New Workhouse Infirmary (W.), 85
Water (P.), 445
Water in Public Baths, The, 3, 50.
Who are the Dirtiest Men? 367
Woman as a Milk Producer, 243
Women, Diseases of, Some Points in the Preventive Treatment of (C.), 27
Workhouse Swill-tub, The, 54
Workmen's Compensation Bill, The, 128, 139, 175
Workmen's Compensation, Its Medical Aspect, 88
Wryneck, Spasmodic, Kocher's Method (P.), 46
X-Ray Dermatitis (P.), 148
"X" Rays. *See also* Roentgen and Röntgen
"X" Rays, The Deep Effects of the, 314
Yellow Fever (C.), 441

### THE SPECIAL SUPPLEMENT FOR NURSES.

À Crete, 24
American Letter, Our, 153, 178
American National Red Cross Society, 193
Antique Safety Pins, 162
Appointments, 11, 19, 25, 34, 42, 50, 61, 78, 88, 96, 113, 124, 134, 146, 149, 166, 172, 177, 184, 195, 203, 217, 228
Appointments, Minor, 6, 20, 26, 34, 42, 50, 59, 70, 87, 100, 106, 108, 118, 127, 131, 146, 152, 166, 172, 175, 183, 194, 202, 209, 218, 225
Asylum Attendants: Their Training and Status, 225
Be Loyal, 97
Book and its Story, A, 41, 98, 210
Book World for Women and Nurses, The, 44, 53, 61, 89, 116, 127, 180, 187
Can a District Nurse be made Self-Supporting? 217
Can the Illiterate and Unphilosophical Rise Superior to the Things that Surround Them? 70
Case for Government Action, A, 18
Con Amore, 219
Cottage in the Country, A, 162
Cyclists and Saddles, 40
Day of Rest for Nurses, 69
Death in our Ranks, 6, 34, 50, 87, 116, 158
Dull Routine of Great Asylums, The, 39
Educational Congress at the Victorian Era Exhibition, 141
Eggs—Nurses, 118
English Nurses in Greece, 87
Etiquette of Nursing, The, 163, 194
Everybody's Opinion, 7, 19, 27, 33, 42, 52, 61, 71, 81, 89, 99, 107, 116, 126, 134, 145, 154, 165, 171, 179, 186, 195, 203, 211, 219, 227
Family Home, A, 115
Feeding the Invalid, 7
For Better or for Worse? 164
Free Guide to Lowestoft, A, 177
Generous Invitation, A, 151
Greek Ladies as Red Cross Nurses, 135
Home and Infirmary for Sick Children, Lower Sydenham, 139
Hospital Etiquette and Discipline, 106
Hospital of the Knights at Malta, The Old, 105,
Hospital Nursing: Where and How to Train, 5
Hospital for Sick Children, The, [illegible]
Hospital Stamps in New South Wales, 185
How to Become a Dispenser, 6
Ideal Nurse, The, 114
Indian Nurses on Active Service, 218
Indian Nursing Service, Some Radical Defects of, 200
Infant Incubators, 151
Importance of Little Things in Nursing, On the, 95
Invalid Cookery, 80
Jubilee Arrangements for Nurses, 89
Jubilee in the African Karoo, 193
Jubilee Keeping in the Vancouver, 202
King of Siam and the Children, The, 184
Lady Army Surgeon, A., 59
Ladies' Waiting Rooms, 88
Lectures to Surgical Nurses, 3, 23, 37, 57, 139, 157, 175, 191, 199, 207, 215
Letter from Corea, A., 209
Letter from Greece, A, 97
Male Nurses at the City Hospital Training School, New York, 227
Matron's Treat, A., 126
Mediæval Practice in the Care of the Sick, 113, 152
Medical Work of the Zenana Mission, The, 136
Men as Nurses, 143
Metropolitan Nursing Association, The, 17
Midwifery, Medical, Surgical, and other Nurses, 126
New Convalescent Home, A, 195
New Hospital for Women, The, 132
News from Greece, 123
News from the Nursing World, 1, 13, 21, 29, 35, 45, 55, 63, 73, 83, 91, 101, 109, 119, 129, 137, 147, 155, 167, 173, 181, 189, 197, 205, 213, 221
Notes and Queries, 12, 20, 28, 34, 44, 54, 62, 72, 82, 90, 100, 108, 118, 128, 146, 154, 166, 172, 180, 188, 196, 204, 212, 220, 228
Novelties for Nurses, 7, 17, 25, 80, 97, 141, 153, 162, 171, 178, 203, 220
Nurses and the Medical Profession, 11
Nurses and Social Grades, 99
Nurses' Co-operation, The, 68
Nurses for Greece, More, 82
Nurses' Hotel, The, 218
Nurses' Inventions, 26
Nurse's Life, Practical Aspects of a, 121
Nurses on Wheels, 49
Nurse's Visit to Texas, A, 60, 78, 123
Nursing in Egypt, 69
Nursing in Shanghai, 226
Nursing, The Etiquette of, 163, 194
Nursing Home for Children, A, 185
Nursing of Phthisical Patients, The, 51
Nursing the Plague in India, 77
Nursing of the Sick in Workhouses, The, 184
Nursing in South Africa, 78
Ormskirk's Intentions, 123
Pensions and Insurances, 218
Pharmacy and Dispensing for Nurses, 15, 31, 47, 65, 75, 85, 93, 103
Plague at Poona, The, 60
Post-Graduate Clinics for Nurses, 4, 16, 32, 38, 48, 58, 66, 76, 86, 94, 104, 112, 122, 132, 140, 150, 158, 170, 176, 192, 201, 208, 216, 224
Power of Influence, The, 151
Power of Judgment, The, 226
Power of Management, The, 133
Practical Aspects of a Nurse's Life, 121, 131, 149, 169, 183, 223
Presentation to Mrs. Coster, 169
Presentation to Miss Philippa Hicks, 185
Presentations, 7, 33, 42, 50, 107, 115, 125, 134, 146, 150, 165, 172, 180, 209, 223
Princess Christian's Trained Nurses, H.R.H., 178
Prince of Wales at Oxford, The, 71
Prize Giving at the Royal Free Hospital, 127
Prizes for Nurses, 107
Probationers: Then and Now, 67
Queen's Day at the National Hospital for the Paralysed and Epileptic, 142
Queen Victoria's Jubilee Institute for Nurses, 133
Queen Victoria's Nurses, 50
Question of Age, A, 59
Reading to the Sick, For, 12, 28, 54, 62, 72, 82, 90, 108, 128, 188, 196, 204, 212, 220, 228
Red Cross and the English Hospitals in Greece, The, 144
Registration of Midwives, The, 77
Rentoul's Will, Dr., 96
Royal British Nurses' Association, 25, 77, 115, 136, 142, 159
Sketches of Hospital Life, 26, 117, 125
Snakes in the Sick Room, 117
Society for the Prevention of Cruelty to Children, The, 177
Surgery at Home, 191
Talks About Food, 124
"The Hospital" Convalescent Fund, 107, 117, 142, 165, 170
Thoughtful Invitation, A, 133
Trained Nurses, H.R.H. Princess Christian's, 178
Useful Hint, A, 215
Verily Mysterious Rays, 226
Victoria Commemoration Club, The, 9, 44, 96, 107, 151
Victorian Era Exhibition, The, 79
Victorian Era Exhibition, Educational Congress at the, 141
Wants and Workers, 24, 34, 42, 50, 78, 90, 116, 131, 146, 152, 166, 201, 219, 227
Wardmaids, On, 211
Warning from the Cape, A, 87
Where To Go, 6, 17, 24, 33, 40, 53, 59, 68, 81, 100, 106, 118, 126, 136, 146, 180, 202, 211
Workhouse Infirmary Association, 81

Printed by W. H. and L. Collingridge, "City Press," 148 and 149, Aldersgate Street, London, E.C.

### EARLY EDITION.

**Notices of Births, Deaths, and Marriages** are inserted in this column at a charge of 2s. 6d. for an announcement not exceeding four lines.

**Notice to Advertisers and Subscribers.**

All Advertisements, Orders for Copies of The Hospital, and Business Communications should be addressed to **THE MANAGER, (Not to The Editor),** The Hospital, The Hospital Building, 28 & 29, Southampton Street, Strand, London, W.C.

The Cost of Subscription, post free, is as follows:—Per week, 2½d.; per quarter, 2s. 8d.; per half-year, 5s. 3d.; per annum, 10s. 6d. Foreign:—Per annum, 15s. 2d.; payable, in all cases, in advance.

**NOTICE.—Vol. XXI. of "The Hospital" (October, 1896, to March, 1897), in handsome cloth binding, will be on sale at the Office of this Journal early in April, price 7s. 6d. Cases for binding the Numbers contained in this Volume, 1s. 6d. Index to Vol. XXI., 2½d., post free.**

**The Monthly Part for April is now ready. Price 6d., by post 8d.**

### NOTES AND NEWS.

### SCRAPS AND GLEANINGS.

PATIENTS' payments to the Samaritan Hospital for Women at Nottingham came to £512 in 1896, out of a total ordinary income of £745. The expenditure amounted to £755. 169 in and 2,532 out patients were treated during the year.

MESSRS. STREET AND Co., advertising agents, of 30, Cornhill, E.C., and 5, Serle Street, W.C., announce that, in consequence of their increasing business and for the convenience of their West-End clients, they will open on Monday, April 5th, 1897, a branch establishment at 164, Piccadilly, London, W.

MR. WILLIAM TALLACK, secretary of the Howard Association, delivered an interesting lecture on "John Howard as a Sanitarian" at the Toynbee Hall recently. Howard's work extended over dwellings, prisons, hospitals, and workhouses.

SINCE the Plymouth Royal Eye Infirmary was established, 75 years ago, 76,103 patients have been treated by it for diseases of the eye. Of these it is stated that 74,684 have either been cured or relieved: 1,069 proved incurable. The patients during 1896 numbered 1,433, of whom 234 were in-patients.

### THE STUDENT OF MEDICINE.

### APPOINTMENTS.

H. A. Collins, M.R.C.S.Eng., L.S.A., appointed Medical Officer for the Saxmundham District of the Plomegate Union. C. Corben, M.R.C S.Eng., L.R.C.P.Lond , appointed Medical Officer for the Caldecott District of the Chepstow Union. G. Cowen, M.D.Dub., M.B , B.Sc., appointed Medical Officer for the Malden District of the Kingston Union. James Crooks, M.D and C.M.-Trin.Toronto, L.R.C.S.Edin., L.S A.Lond., appointed Medical Officer and Public Vaccinator for the Eighth District of the Lexden and Winstree Union, and the Seventh District of the Sudbury Union. E. L. Davey, M.R.C.S.Eng., L.R.C.P.,Lond., appointed Medical Officer for the Third District of the Dover Union. T. M. Day, M.R.C.S.Eng , L.R.C.P Lond., appointed Medical Officer for the Harlow District of the Epping Union. John Eaton, M.D.Glasg., M.B., C.M., appointed Medical Officer and Public Vaccinator for the Cleator District of the Whitehaven Union. Dr. A. Edwards, appointed House Surgeon to the Greenock Infirmary. F. M. Fellows, M B., C.M.Edin., appointed House-Surgeon to the General Hospital, Great Yarmouth. Stephen Floyd, M.D., appointed Medical Officer of Health to the Llandrindod Wells Urban District Council. R. Hutchison, M.D., appointed Demonstrator of Physiology at the London Hospital Medical College. J. Iredale, L.R.C.P., L.R.C S.Edin., appointed Medical Officer of Health to the Mablethorpe Urban District Council. J. P. Johnstone, L.R.C.P., L.R.C.S.Edin., appointed Medical Officer for the First A District of the Langport Union. E. P. Manby, B.A., M.D., D.P.H., appointed Assistant Lecturer on Public Health at University College, Liverpool. Dr. H. Marshall, appointed Medical Officer for the Third District of the Battle Union. W. Millar, M.B., L.R.C.P., L.R.C.S.Edin., appointed Medical Officer for the Wilton District of the Sculcoates Union. M. P. Norman, M.D.Dub., B.Sc., appointed Medical Officer of the Workhouse and the Coddenham District of the Bosmere and Claydon Union. G. P. O'Connor, L.R C.P., L.R.C.S.Edin., appointed Medical Officer for the Fourth District of the North Witchford Union. T. H. Parke, M.R.C S.Eng., L.R.C.P.Edin., L.S.A., appointed Medical Officer for the Tideswell District of the Bakewell Union. F. G. M. Philps, M.R.C.S., L.R.C P., appointed Clinical Assistant to out-patients at the Chelsea Hospital for Women. Harold J. Pickering, L.D S.R C S.-Eng , appointed Dental Surgeon to the Felstead School, Essex. J. E. Pollard, L.R.C.P., L.R.C.S.Edin., L.F.P.S Glasg., appointed Medical Officer for the Third District of the Dover Union. G. Basil Price, M.B., B.S.Lond , L.R.C.P., M.R.C.S.-Eng , appointed Resident Medical Officer to the London Temperance Hospital, Hampstead Road, N.W. John Richards, M.B.-Edin., appointed Assistant Medical Officer in the Derby Borough Asylum. Mr. Rimell, appointed Medical Officer for the Tydd, Sutton St. James, and Long Sutton Districts of the Spalding Union. Robert M. Simon, M.D Cantab., F.R.C.P., appointed to the Professorship of Forensic Medicine at Mason College, Birmingham. R A. Skinner, M.R.C.S.Eng., appointed Medical Officer for the Tenterden District and Workhouse of the Tenterden Union. A. J. A Theobald, M.B.Edin., appointed Medical Officer for the Reading and Wokingham School District. John A. Turner, M.B.Edin., appointed Medical Officer of Health for the Buntingford, Hadham, Hertford, Stansted, and Ware Rural Districts, and the Bishop's Stortford, Hertford, Hoddesdon, and Ware Urban Districts. Wm. John H. Wood, L.R.C.P., L.R C.S.-Edin., appointed Medical Officer for the Skirbeck District of the Boston Union.

